# Supplementary material for: Improvement of Sexual Function and Sleep Quality in Patients with Atopic Dermatitis Treated with Dupilumab: A Single-Centre Prospective Observational Study
Source: Int J Environ Res Public Health. 2023 Jan 20;20(3):1918. doi: 10.3390/ijerph20031918 (PMC9914896; doi:10.3390/ijerph20031918)
Supplement: Supplementary file 1 [file ijerph-20-01918-s001.zip › ijerph-2107236-supplementary.pdf]

Table S1. Sociodemographic and clinical characteristics grouped by gender.

	Total	Male	Female	p-value
	(n=32)	(n=12)	(n= 20)	
Age (years)	31.81 (13.93)	30.71 (19.54)	30.4 (14.48)	0.47
Marital status				0.2
Single	68.8% (22)	75% (9)	65% (13)	-
Married	9.4% (3)	8.3% (1)	10%(2)	-
Divorced	9.4% (3)	0%	15% (3)	-
Smoking habit (yes)	9.4% (3)	8.3% (1)	10%(2)	0.93
Alcohol consumption (yes)	28.1% (9)	25% (3)	30% (6)	0.79
Topical corticosteroid use	65.6% (21)	58.3 %(7)	70% (14)	0.54
Frequency of topical corticosteroid use (>4 times/week)	24% (8)	16.3% (14)	30% (8)	0.93
Family history of AD	28.1% (9)	33.3% (4)	25% (5)	0.46
Atopic march symptoms	56.3% (18)	58.3%(7)	55%(11)	0.65
Previous AD treatment (yes)	100% (32)	100%(12)	100%(20)	0.21
Systemic treatment	93.8% (30)	91.7% (11)	95% (19)	0.45
Biologic treatment	3.1 (1)	8.3%(1)	0%(0)	0.2
Basal EASI	23.24 (6.74)	23.72(8.24)	22.95(5.88)	0.76
Final EASI	7.37 (6.24)	10.46(7.99)	5.74(4.54)	0.51
EASI change	-16.06(7.35)	-14.1(8.18)	-17.09(6.88)	0.36
Basal SCORAD	54.07 (13.89)	50.56(18.62)	55.86(14.47)	0.38
Final SCORAD	28.45 (12.8)	30.28(13.2)	25.95(14.08)	0.45
SCORAD change	-25.62(18.18)	-23.21(19.22)	-26.9(18.08)	0.63
Basal PSQI	7.73(3.69)	11.55(4.08)	13(4.81)	0.41
Final PSQI	12.47(4.54)	8.5(3.25)	7.39(3.91)	0.5
PSQI change	-5.08(4.59)	-3.63(4.44)	-5,76(4.63)	0.29

Data are expressed as mean (standard deviation) or relative (absolute) frequency. AD, atopic dermatitis.
compare qualitative variables.

Figure S1. Individual therapeutic responses regarding Eczema Area and Severity (EASI) (a), Pittsburgh Sleep Quality Index (PSQI) (b), Female Sexual Function Index (FSFI) (c) and International Index of Erectile Dysfunction 5 (IIEF-5) (d) in week 0 and 16 after treatment with dupilumab.

a.

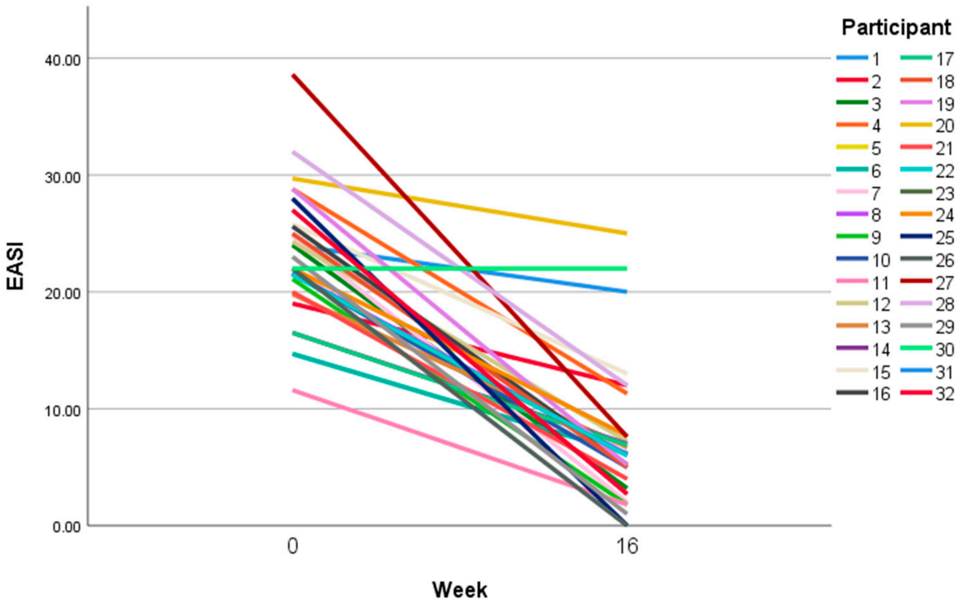

b.

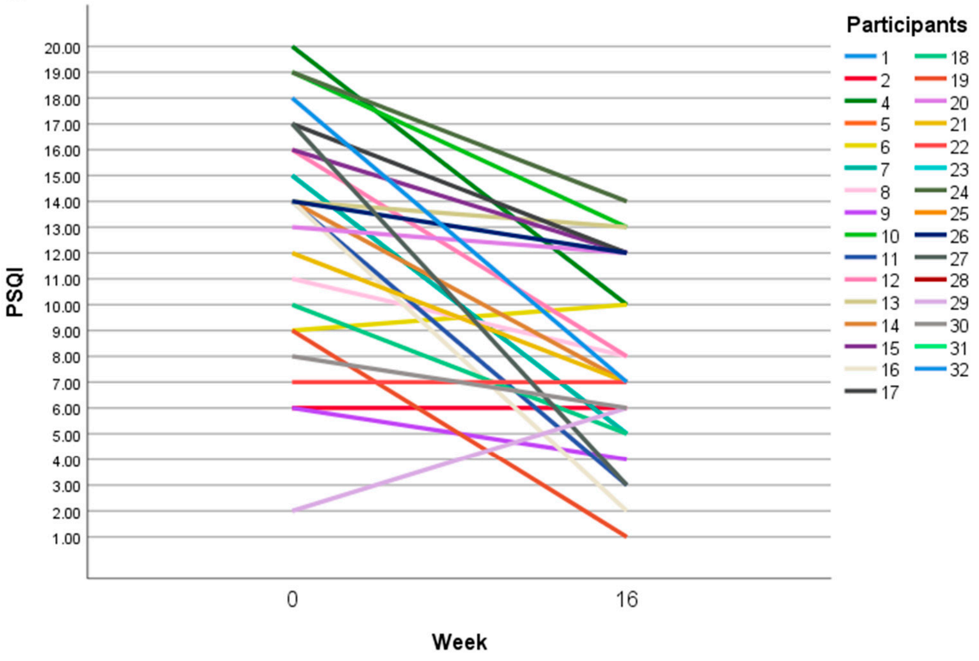

c.

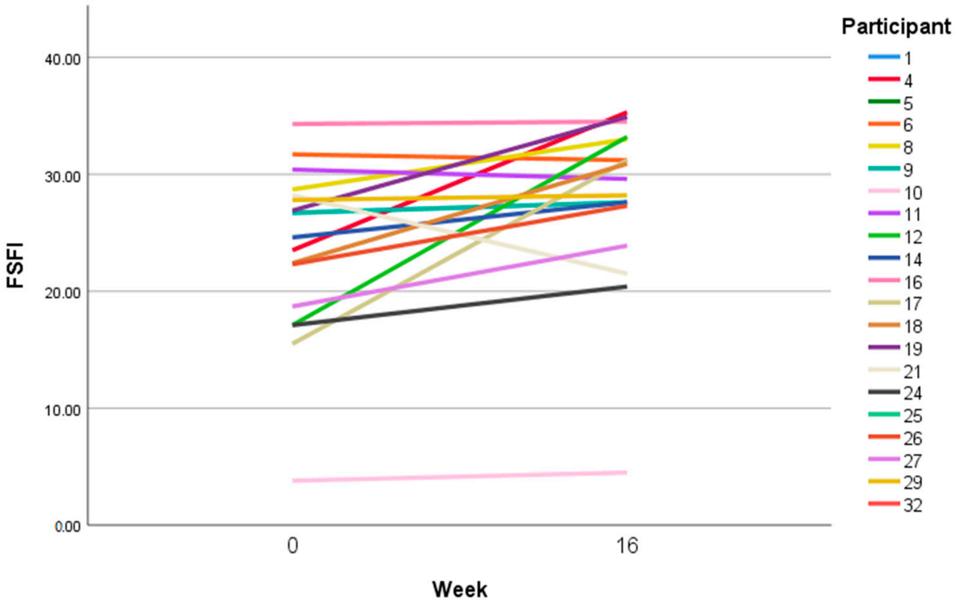
Participant
1
4
5
6
8
9
10
11
12
14
16
17
18
19
21
24
25
26
27
29
32
FSFI
40.00
30.00
20.00
10.00
0.00
0
16
Week

d.

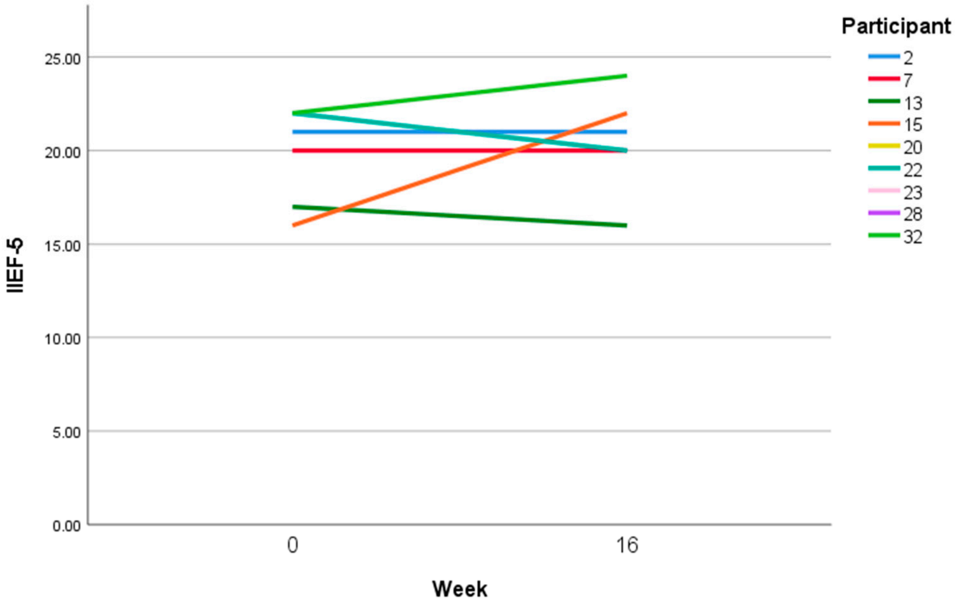
Participant
2
7
13
15
20
22
23
28
32
IIEF-5
25.00
20.00
15.00
10.00
5.00
0.00
0
16
Week
